# Supplementary material for: Emergence of crucial evidence catalyzing the origin tracing of SARS-CoV-2
Source: PLoS One. 2024 Aug 30;19(8):e0309557. doi: 10.1371/journal.pone.0309557 (PMC11364235; doi:10.1371/journal.pone.0309557)
Supplement: Attachment — Submitted filename: Editor PLOSONE.docx [file pone.0309557.s002.docx]

Editor, Dated: 25/06/2024

**PLOSONE**

**Reference No**. :- PONE-D-24-20443
**Title**:- Emergence of crucial evidence catalyzing the origin tracing of SARS-CoV2
Greetings,

From my observation, the bioinformatics-related research article titled, “Emergence of crucial evidence catalyzing the origin tracing of SARS-CoV-2” makes no sense and is devoid of any novelty. This article has been written in a very bizarre manner. I am not able to understand what they want to convey. To my understanding, I have observed the below mentioned flaws. These are as follows:-

1. The abstract of this manuscript comprises of many mistakes such as, BANAL-20-52 Belong to. Belong to what?
2. The three strains of the SARS-CoV2 namely; BANAL-20-52, Rp22DB159, and S18CXBatR24 have been already identified.
3. One can’t claim purely on the basis of bioinformatics that we reject the laboratory-based engineering of SARS-CoV2 harboring furin-cleavage site between S1 and S2 subunit of the SARS-CoV2 spike protein.
4. Introduction section of this article has been written poorly with no coherence in order to comprehend it effectively and objectively.
5. Sentence under the Line number 54 and 55 is totally incorrect in terms of calculating the percentage of nucleotide identity.
6. References have been inserted within the sentences and sound very bad.
7. You can’t write, “Unfortunately, our research proposal was not funded”.
8. Do you think that readers have that much time to go through the Figure No. 3?
9. One can’t write materials and methods in result section.
10. In sentence number 423-424, you write unfortunately the genetic and geographical origin of SAR-COV2 remains unknown, resulting in suspicion about its origin. This sentence is contrary to your statement in the abstract section.

Overall, I am not happy with the findings, result section, and figures. I personally cannot recommend this paper for its possible consideration in PLOSONE journal.

Wishing you all the best

********************************************************************

Dr Arif Bashir

Assistant Professor & Head, Biotechnology
