# Supplementary material for: Emergence of crucial evidence catalyzing the origin tracing of SARS-CoV-2
Source: PLoS One. 2024 Aug 30;19(8):e0309557. doi: 10.1371/journal.pone.0309557 (PMC11364235; doi:10.1371/journal.pone.0309557)
Supplement: Attachment — Submitted filename: Response to Reviewers.doc [file pone.0309557.s003.doc]

# Point to point responses

**Dear Dr. AbdulAzeez Adeyemi Anjorin and the editorial**

Thank you very much for your help on the reviewing of this paper. The reviewer's comments are very professional and helped us on the improvement of this manuscript. Following the reviewers’ suggestions, we revised the manuscript and uploaded the revised version. Although we note that Reviewer 3 raised concerns that extend beyond the academic scope and reflect a certain bias, we still did our utmost to address his concerns.

Shan Gao, PhD

Associate Professor

College of Life Sciences

Nankai University

http://sky.nankai.edu.cn/english/teacher.asp?id=118

ORCID: 0000-0002-8919-1338

**Reviewer's Comments:**

**Reviewer #1:**

Chen S et al., analyzed the phylogenetic relationships among the Sarbecoviruses (in genus Betacoloronavirus) detected before outbreak of SARS-CoV-2 in the end of 2019 to trace the origin of SARS-CoV-2 in natural reservoir. The authors used 292 nucleotide sequences of Sarbecoviruses in Genbank including 156 bat-origine Sarbecoviruses for analysis.

There have been numerous epidemiological studies to find the origin of SARS-CoV2, but no clear conclusion has been reached. This study is unique in that it focuses on the nine genetic markers ‘RC0 - RC7 and ORF8’ rather than sequence similarity of whole gene taking into the consideration of frequence recombination of coronavirus genome.

Although the study did not reach out the direct origin of coronavirus strain connected to SARS-CoV-2, the analysis proposed that RC1, one of the genetic markers would contribute to trace the history of Sarbecoviruses in wild life.

**Response:** Thank you for your comments and suggestions. We have provided point-by-point responses to address your concerns. If these do not fully clarify your questions, please let us know, and we will be happy to offer further explanations. By the way, I would like to express my sincere appreciation for your invaluable assistance in the publication of our research. Your contribution will significantly aid in origin tracing of SARS-CoV-2.

Minor comment

Throughout the manuscript, descriptions seem subjective. It is recommended to describe result and discussion separately to differentiate the objective facts clearly.

**Response**: thank you, I did some modification.

Table 1. What are the numbers in the table?

**Response**: InDel represents insertions or deletions, while the number behind InDel indicates how many aa residues have been substituted.

Abstract. Please delete or modify the last sentence.

**Response**: it was deleted.

Is it reasonable to treat gene deletions and insertions equally?

**Response**: No, but we have no alternative choices. Without prior knowledge, we have to treat each mutation equally.

Line 135. Please explain the RC0 ‘e.g. addition R insertion at position XX-XX’ here again.

**Response**: they were explained in the legend of figure 1, and in the text, we inserted a “figure 1”. We omitted an explanation in the text to prevent distracting the readers.

Fig. 1. The position and scale of S1-S2 gene showing RC3-RC7 and RC0 (shown in right-bottom) was difficult to understand. Please make clear the relationship to the image of SARS-CoV-2 gene structure.

**Response**: it was modified.

Line 136-138. Please explain the example of genetic characteristics of ORF8.

**Response**: a reference was inserted there to explain the example of genetic characteristics of ORF8.

Lines 141-145. Why was RC8 was not included in the analysis? If not used for analysis, please consider to remove the description of RC8 as it would be confusing.

**Response**: RC8 does not exhibits diversity as the other RCs, so it was not used for the analysis.

Lines 208-209. Please explain the author’s criteria here to remove RaTG13.

**Response**: the criteria was described in Materials and Methods, and we omitted an explanation in the text to prevent distracting the readers. Here it is,

Close relatives of SARS-CoV-2 were identified according to the criteria: a toal of 343-aa residues of peptides or protein segments encoded by the nine key genomic features are required to be 100% covered with at least 90% identitiy.

Fig. 2. Assign Fig. 2A, B, D.

Fig. 2. Provide amino acid and nucleotide numbers.

**Response**: it was modified.

Lines 428-430. Generations of gene-engineered virus do not rely on particular strain. This claim here seems unworthy of your excellent work.

**Response**: thank you very much for your kind consideration. The old sentence was removed to avoid some “troubles”. I added a new one “As Feuang of Laos is the sole place where eight of the nine wild-type features (RC1-7 and ORF8) have been detected, it is considered more probable than Wuhan to be the initial site where the ancestor of SARS-CoV-2 was transmitted from bats to intermediate hosts or directly to humans. The new sentences are more objective.

**Reviewer #2:**

Dear PLOS ONE Editor

I accepted to review this article as I found this paper to be very interesting and worth to be published , however after including the structural mechanism to block Covid 19 infection which is the receptor binding domain (RBD) and the junction furin cleavage site(FCS) between S1 and S2, as stated in the paper. Mentioning the structural features required to block infection at these positions will strengthen the paper.

I suggest at the end of Results and Discussion paper to add a sub section paragraph referring to the RBD/ ACE2 and FCS mechanisms which invoke infection and relevant drug therapies, like angiotensin receptor blockers (ARBs) that block infection to enhance the strength of paper.

In particular,

The authors should mention the infection mechanism and ARBs potency in blocking virus infection using RBD /ACE2 and FCS as targets based on anionic structural features, like carboxylates and tetrazolates, required for inhibition and described in following papers suggested to be cited. This could be then a much useful paper for the scientific community.

Specifically, ARBs and bisartans with their negative structural features- warheads as tetrazolates in angiotensin receptor blockers (ARBs)- can block targets like RBD:ACE2 containing arginines and FCS by complexing with positive arginines at arginine rich multi basic sites S1/S2 (680-686) and S2(810-816) of furin which catalyse the cleavage of spike protein initiating infection. ARBs and especially bisartans with two tetrazolates inactivate the cleavage of furin by complexing to arginines, thus preventing infection. This is a key point in the mechanism of infection/ inhibition and worth to be mentioned. This will increase credibility and value of the paper.

**Response:** Thank you for your comments and suggestions. We have provided point-by-point responses to address your concerns. If these do not fully clarify your questions, please let us know, and we will be happy to offer further explanations. By the way, I would like to express my sincere appreciation for your invaluable assistance in the publication of our research. Your contribution will significantly aid in origin tracing of SARS-CoV-2.

Relevant papers to be cited

1. Ridgway et al, Expert Opinion on Therapeutic Targets, 1-23, 2024, DOI( available)

2. Ridgway et al Computational and Structural Biotechnology Journal, 20, 2091, 2022

3. Ridgway et al, Viruses, 14, 1029, 2022

4. Ridgway et al, Computational and Structural Biotechnology Journal, 21, 4589, 2023

5. Ridgway et al, Viruses, 15, 309, 2023.

6. Swiderski et al , Biomolecules, 13, 787, 2023

7. Kelaidonis et al, International Journal of Molecular Sciences, 24, 8454, 2023

8. Moore et al , Molecules, 27, 4854,2022.

9. Moore et al, Biomolecules, 11, 979, 2021

Authors should also mention in Conclusion Section, in a few words, the structural role in the infection- protection process.

**Response:** To smoothly transit to mentioning the structural features, we added some sentences in the Conclusion Section, “In addition, the structural features of the protein regions encoded by RC0 and RC1 could be used in drug design to inhibit or even block the infection of SARS-CoV-2. For instance, angiotensin receptor blockers (ARBs) have been designed”

And we cited 7 of 9 papers as [27-31]

27.	Ridgway H, Moore GJ, Gadanec LK, Zulli A, Apostolopoulos V, Hoffmann W, et al. Novel benzimidazole angiotensin receptor blockers with anti-SARS-CoV-2 activity equipotent to that of nirmatrelvir: computational and enzymatic studies. Expert Opin Ther Tar. 2024;28(5): 437-459.

28.	Swiderski J, Gadanec LK, Apostolopoulos V, Moore GJ, Kelaidonis K, Matsoukas JM, et al. Role of angiotensin II in cardiovascular diseases: Introducing bisartans as a novel therapy for coronavirus 2019. Biomolecules. 2023;13(5): 787.

29.	Ridgway H, Chasapis CT, Kelaidonis K, Ligielli I, Moore GJ, Gadanec LK, et al. Understanding the driving forces that trigger mutations in SARS-CoV-2: mutational energetics and the role of arginine blockers in COVID-19 therapy. Viruses. 2022;14(5): 1029.

30.	Ridgway H, Moore GJ, Mavromoustakos T, Tsiodras S, Ligielli I, Kelaidonis K, et al. Discovery of a new generation of angiotensin receptor blocking drugs: Receptor mechanisms and in silico binding to enzymes relevant to SARS-CoV-2. Comput Struct Biotec. 2022;20: 2091-2111.

31.	Moore GJ, Pires JM, Kelaidonis K, Gadanec LK, Zulli A, Apostolopoulos V, et al. Receptor interactions of angiotensin II and angiotensin receptor blockers-relevance to COVID-19. Biomolecules. 2021;11(7): 979.

After addressing the above, I agree and recommend this nice research to be published as an important contribution to the field and science.

John Matsoukas

Professor of Chemistry

Honorary Professor

**Reviewer #3:**

Reference No. :- PONE-D-24-20443

Title:- Emergence of crucial evidence catalyzing the origin tracing of SARS-CoV2

Greetings,

From my observation, the bioinformatics-related research article titled, “Emergence of crucial evidence catalyzing the origin tracing of SARS-CoV-2” makes no sense and is devoid of any novelty. This article has been written in a very bizarre manner. I am not able to understand what they want to convey. To my understanding, I have observed the below mentioned flaws. These are as follows:-

**Response:** Thank you for your comments and suggestions. We have provided point-by-point responses to address your concerns. If these do not fully clarify your questions, please let us know, and we will be happy to offer further explanations. I am not sure whether these responses can make you happy or not, but I will do my utmost to address your concerns.

1. The abstract of this manuscript comprises of many mistakes such as, BANAL-20-52 Belong to. Belong to what?

**Response**: “The most significant contribution of the present study lies in the detection of a key genomic feature, designated as RC1, in wild genotype in a bat Sarbecovirus population BANAL-20-52 belonging to.” Belong to a bat Sarbecovirus population. I donot think there is any mistake in this sentence.

2. The three strains of the SARS-CoV2 namely; BANAL-20-52, Rp22DB159, and S18CXBatR24 have been already identified.

**Response**: The three strains of the SARS-CoV2 namely; BANAL-20-52, Rp22DB159, and S18CXBatR24 have been already identified as belonging to bat Sarbecovirus. Then, they were identified as three close relatives in our study. By the way, SARS-CoV2 has been already identified and researchers are still studying it.

3. One can’t claim purely on the basis of bioinformatics that we reject the laboratory-based engineering of SARS-CoV2 harboring furin-cleavage site between S1 and S2 subunit of the SARS-CoV2 spike protein.

**Response**: Even if the laboratory-based engineering strain harboring furin-cleavage site was produced, it can not evolve into SARS-CoV2 without the other eight wild-type features.

As Feuang of Laos is the sole place where eight of the nine wild-type features (RC1-7 and ORF8) have been detected, it is considered more probable than Wuhan to be the initial site where the ancestor of SARS-CoV-2 was transmitted from bats to intermediate hosts or directly to humans.

4. Introduction section of this article has been written poorly with no coherence in order to comprehend it effectively and objectively.

**Response**: please point out mistakes as question 1, however, that is not a mistake.

5. Sentence under the Line number 54 and 55 is totally incorrect in terms of calculating the percentage of nucleotide identity.

**Response**: what are the correct ones? 28940/29881=96.85%? 28720/29875=96.13%?

6. References have been inserted within the sentences and sound very bad.

**Response**: References were placed in close proximity to the related concepts, enhancing their accessibility for readers

7. You can’t write, “Unfortunately, our research proposal was not funded”.

**Response**: this answered your question why we cannot afford experiments. Now, you understand how hard we are trying to do contribution for the orgin tracing of SARS-CoV-2.

8. Do you think that readers have that much time to go through the Figure No. 3?

**Response**: figure 3 like figures are often used in Phylogeography.

9. One can’t write materials and methods in result section.

**Response**: the section of materials and methods was after the section of results. Now, it was changed, according to the plosONE requirements.

10. In sentence number 423-424, you write unfortunately the genetic and geographical origin of SAR-COV2 remains unknown, resulting in suspicion about its origin. This sentence is contrary to your statement in the abstract section.

**Response**: that is why we provided RC1 as a new clue.

Overall, I am not happy with the findings, result section, and figures. I personally cannot recommend this paper for its possible consideration in PLOSONE journal.

Dr Arif Bashir

Assistant Professor & Head

Department of Biotechnology & Clinical Biochemistry

Government College for Women, Nawa-Kadal

J&K-India
